# Silk fibroin-based hydrogels for cartilage organoids in osteoarthritis treatment

**DOI:** 10.7150/thno.103491

**Published:** 2025-01-01

**Authors:** Congyi Shen, Ziyang Zhou, Ruiyang Li, Shike Yang, Dongyang Zhou, Fengjin Zhou, Zhen Geng, Jiacan Su

**Affiliations:** 1 Institute of Translational Medicine, Shanghai University, Shanghai, 200444, China.; 2 Organoid Research Center, Shanghai University, Shanghai, 200444, China.; 3 National Center for Translational Medicine (Shanghai) SHU Branch, Shanghai University, Shanghai, 200444, China.; 4 School of Medicine, Shanghai University, Shanghai, 200444, China.; 5 Department of Orthopedics, Xinhua Hospital, Shanghai Jiao Tong University School of Medicine, Shanghai, 200092, China.; 6 Department of Anesthesiology, Shanghai Zhongye Hospital, Shanghai, 200941, China.; 7 Department of Orthopedics, Honghui Hospital, Xi'an Jiao Tong University, Xi'an, 710000, China.

**Keywords:** Osteoarthritis, Silk fibroin, Hydrogels, Cartilage organoids, Cartilage regeneration.

## Abstract

Osteoarthritis (OA) is a common joint disease characterized by cartilage degeneration. It can cause severe pain, deformity and even amputation risk. However, existing clinical treatment methods for cartilage repair present certain deficiencies. Meanwhile, the repair effect of cartilage tissue engineering is also unsatisfactory. Cartilage organoids are multicellular aggregates with cartilage-like three-dimensional structure and function. On the one hand, cartilage organoids can be used to explore the pathogenesis of OA by constructing disease models. On the other hand, it can be used as filler for rapid cartilage repair. Extracellular matrix (ECM)-like three-dimensional environment is the key to construct cartilage organoids. Silk fibroin (SF)-based hydrogels not only have ECM-like structure, but also have unique mechanical properties and remarkable biocompatibility. Therefore, SF-based hydrogels are considered as ideal biomaterials for constructing cartilage organoids. In this review, we reviewed the studies of cartilage organoids and SF-based hydrogels. The advantages of SF-based hydrogels in constructing cartilage organoids and the iterative optimization of cartilage organoids through designing hydrogels by using artificial intelligence (AI) calculation are also discussed. This review aims to provide a theoretical basis for the treatment of OA using SF-based biomaterials and cartilage organoids.

## 1. Introduction

Osteoarthritis (OA) is a joint disease characterized by cartilage degeneration 1-5. Pathogenic factors of OA are mainly associated with obesity and age 6. As the global aging and obese population rises, the number of OA patients will increase sharply in the next decade 7, 8. Statistically, approximately 595 million people worldwide are affected by OA. And by 2050, the prevalence is projected to increase by over 200%, resulting in substantial economic burdens on both individuals and society 9. Cartilage degeneration plays a significant role in the progression of OA, as it contributes not only to the disease outcome but also to its worsening 10, 11. Therefore, repairing cartilage is a valuable strategy for the treatment and prevention of OA.

Cartilage has limited self-repair capacity 12-14. As an immune-privileged tissue, it makes engineered cartilage analogs attractive candidates for off-the-shelf grafts in allogeneic transplantation. Organoids are simplified multicellular structures that develop from stem cells or organ progenitors through *in vitro* 3D culture combined with targeted induction technology, enabling the formation of organ-specific architectures and functions 15-17. Specifically, cartilage organoids are tissues with cartilage structure, function, physiological and pathological characteristics through cultivating and assembling stem cells or chondrocytes 18, 19. This offers a novel approach for cartilage regeneration. Compared to autologous chondrocyte implantation (ACI), cartilage organoid transplantation eliminates the need for a secondary surgery to harvest cells, streamlining the procedure and reducing postoperative complications. Techniques like ACI and matrix-induced autologous chondrocyte implantation (MACI), which rely on 2D chondrocyte expansion, are time-consuming and increase the risk of dedifferentiation into fibrocartilage 20-22. In contrast, cartilage organoids have distinct advantages: they replicate the natural cartilage structure and can be directly implanted to repair defects, ensuring that the regenerated tissue closely resembles native cartilage. Furthermore, as they already mimic cartilage properties, cartilage organoids can seamlessly integrate with native tissue, minimizing the need for further regeneration post-implantation and thus enhancing the overall repair efficiency 23.

Organoid construction methods are broadly divided into scaffold-free self-organization and biomaterial-based co-cultivation. Scaffold-free self-organization allows mesenchymal stem cells (MSCs) to naturally form complex tissue structures. This method is straightforward and has a lower risk of contamination, but it struggles with achieving consistent organoid size and uniformity. On the other hand, biomaterial-assisted co-cultivation uses scaffolds like hydrogels to provide structural support, allowing precise control over the organoid architecture and customization of the environment. This makes it ideal for developing organoids with consistent and predictable properties. Currently, Matrigel is the most commonly used material for organoid construction. However, its undefined composition and batch-to-batch variability result in inconsistent mechanical strength, making it less reliable for precise experimental needs. Additionally, Matrigel lacks the flexibility for customization in specific organoid culture contexts 18, 24, 25. Silk fibroin (SF) hydrogels, as a natural macromolecular material, offer several advantages over Matrigel, including a well-defined structure, controllable mechanical properties, and high customizability. These features make SF hydrogels more suitable for precise tissue engineering applications 26-30. Moreover, SF's excellent processability allows it to be adapted to various processing methods and functional modifications, making it versatile for preparing different types of cartilage tissue engineering materials 31-33. Furthermore, SF hydrogels have excellent printability, enabling their use in 3D bioprinting for advanced biofabrication techniques 34-36. Therefore, SF-based hydrogel has a great prospect for cartilage regeneration and cartilage organoids construction 37.

**Figure 1:**
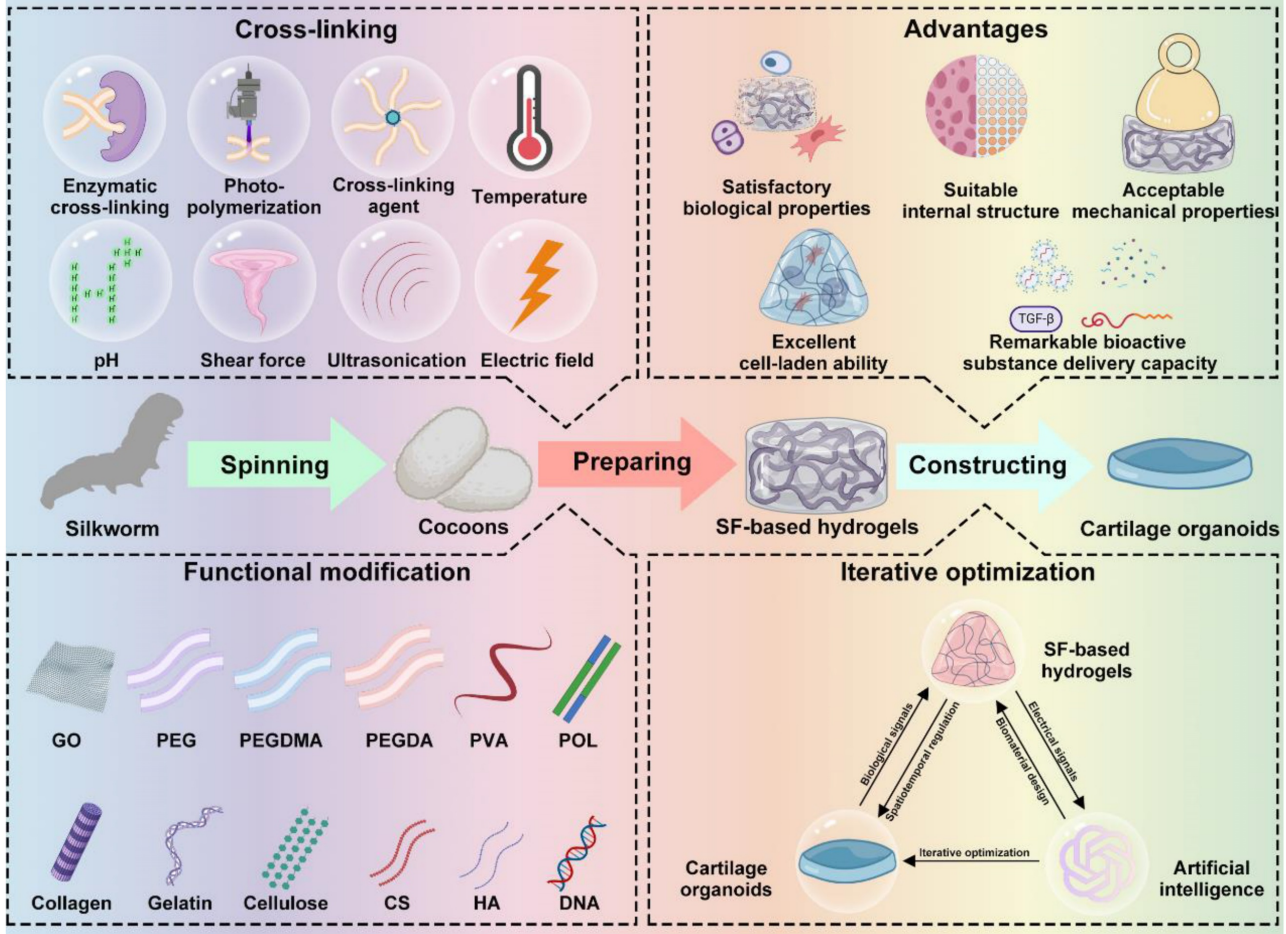
Schematic diagram of SF-based hydrogels for construction of cartilage organoids. Created with BioRender.com.

Herein, we summarize recent research on cartilage organoids and SF-based hydrogels, highlighting the advantages of SF-based hydrogels for cartilage organoid construction. The iterative optimization of cartilage organoids through designing hydrogels by using artificial intelligence (AI) calculation is also discussed (**Figure 1**). We hope that this review can provide a reference for cartilage organoids construction and a promising therapeutic strategy for OA.

## 2. Research progress in cartilage organoids

**Figure 2:**
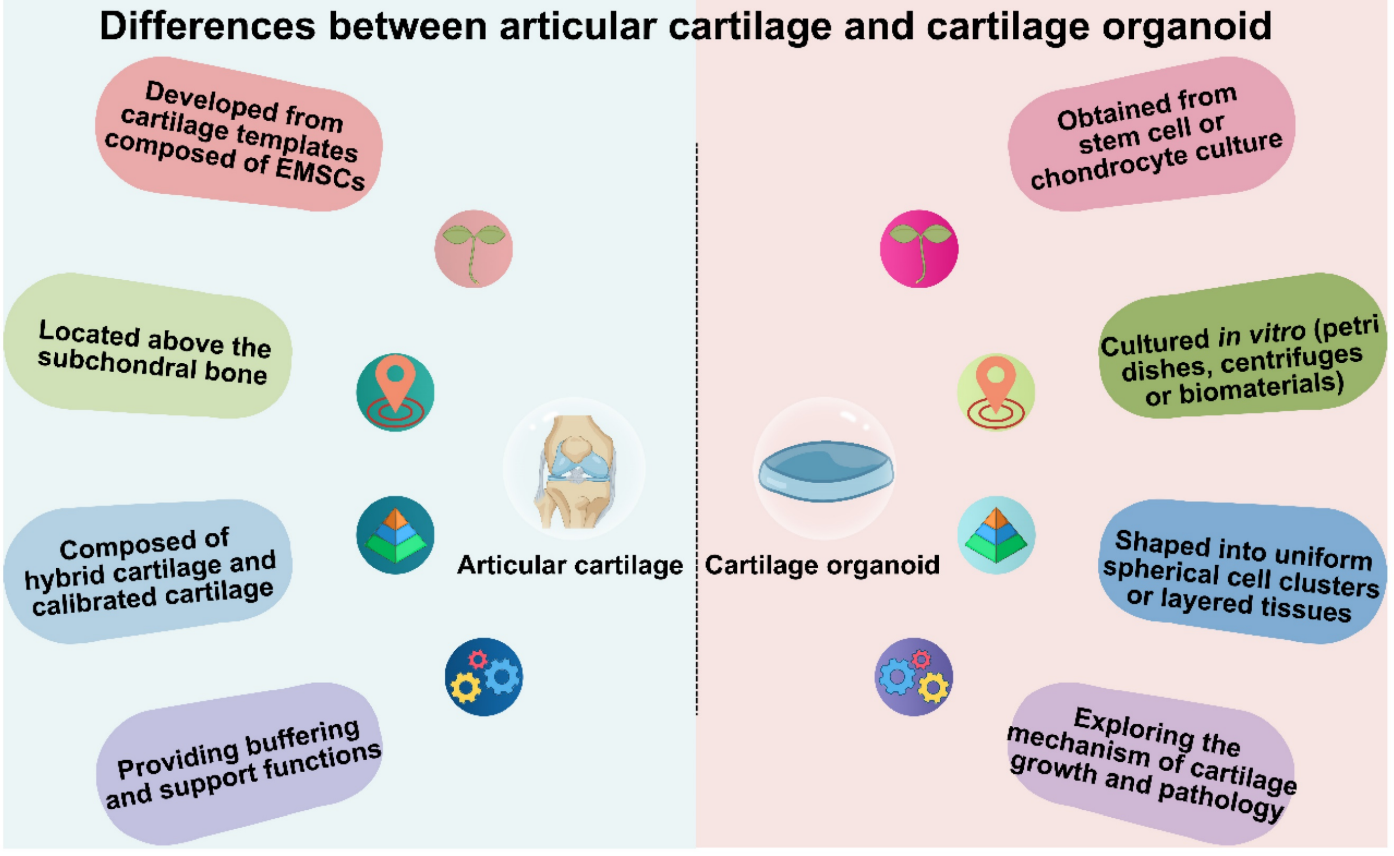
Differences between articular cartilage and cartilage organoids in origin, location, structure, and function. Created with BioRender.com.

**Figure 3:**
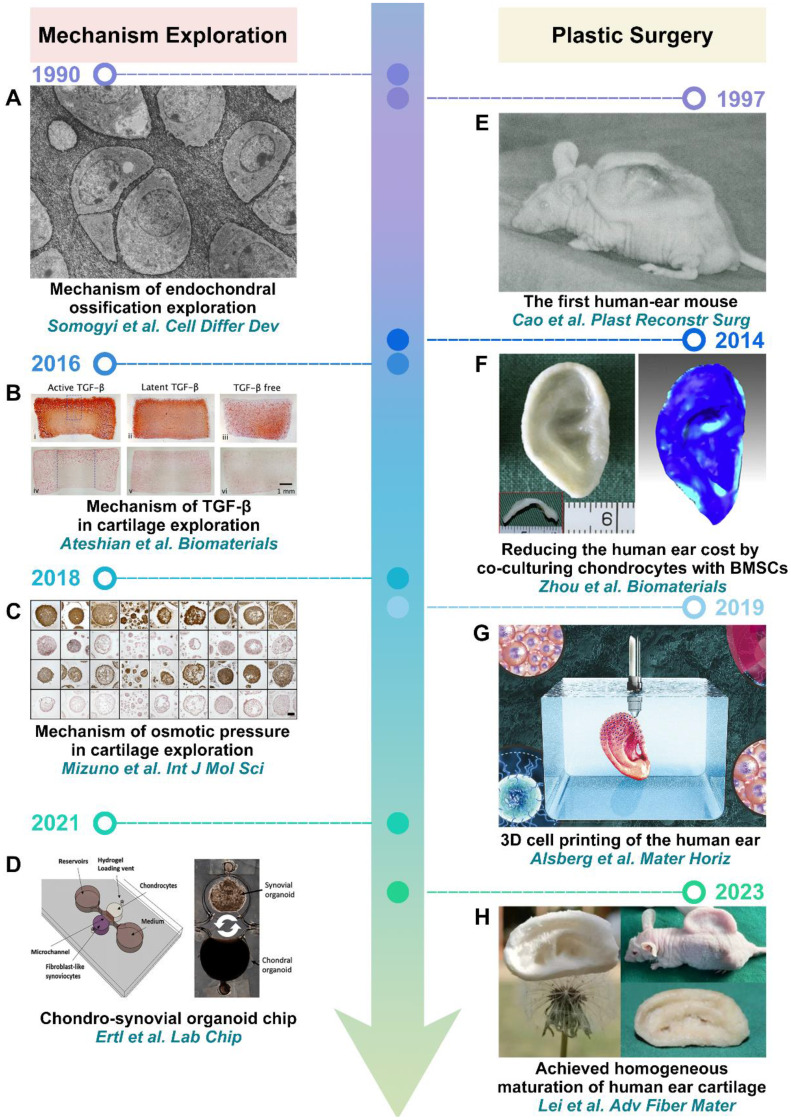
** Cartilage organoids in mechanism exploration and plastic surgery: (A)** Reproduced with permission from ref 43; Copyright 1990, Elsevier. **(B)** Reproduced with permission from ref 44; Copyright 2016, Elsevier. **(C)** Reproduced with permission from ref 45; Copyright 2018, MDPI. **(D)** Reproduced with permission from ref 46; Copyright 2021, Royal Society of Chemistry. **(E)** Reproduced with permission from ref 48; Copyright 1997, Wolters Kluwer Health, Inc. **(F)** Reproduced with permission from ref 49; Copyright 2014, Elsevier. **(G)** Reproduced with permission from ref 50; Copyright 2019, Royal Society of Chemistry**. (H)** Reproduced with permission from ref 51; Copyright 2023, Springer Nature.

**Figure 4:**
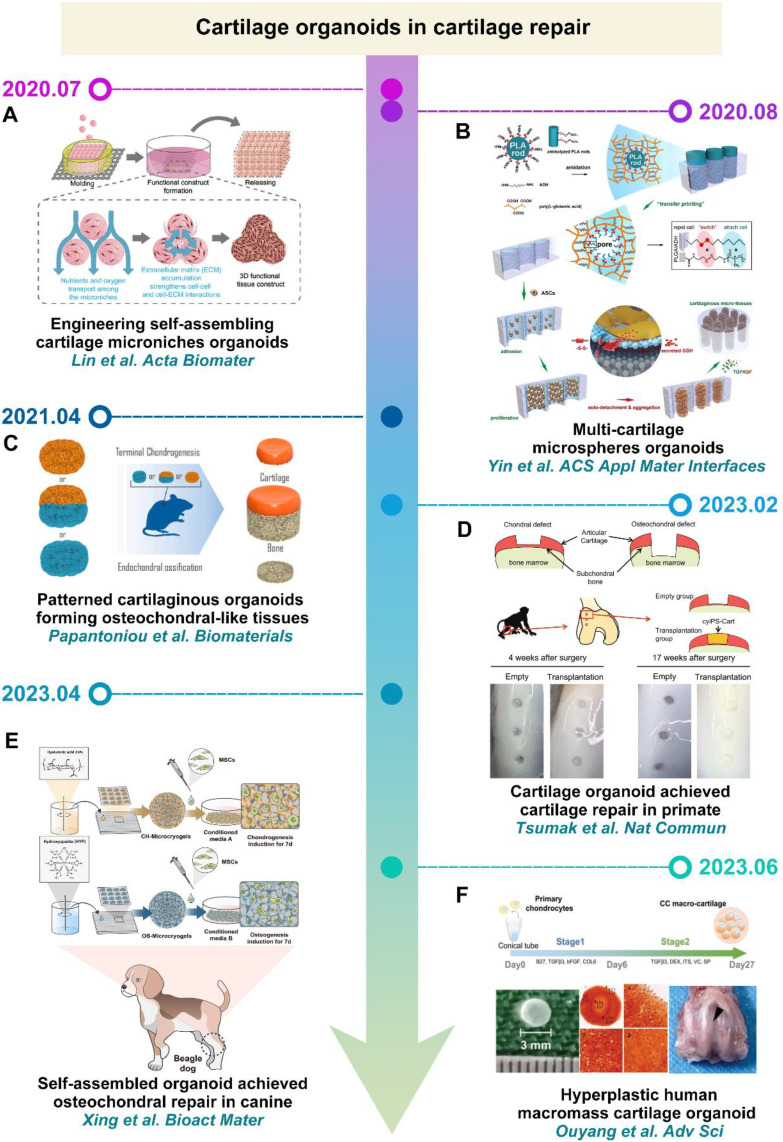
**Cartilage organoids in tissue engineering: (A)** Reproduced with permission from ref 52; Copyright 2020, Elsevier. **(B)** Reproduced with permission from ref 53; Copyright 2020, American Chemical Society. **(C)** Reproduced with permission from ref 54; Copyright 2021, Elsevier. **(D)** Reproduced with permission from ref 55; Copyright 2023, Springer Nature. **(E)** Reproduced with permission from ref 56; Copyright 2023, Elsevier. **(F)** Reproduced with permission from ref 57; Copyright 2023, Wiley-VCH GmbH.

Cartilage is a critical component of the human body, providing essential support for mechanical reinforcement, cushioning, and protection (**Figure 2**). Cartilage development commences with a cartilaginous template constituted by embryonic mesenchymal stem cells. During this stage, primitive embryonic mesenchymal cells initiate differentiation into chondroblasts 38-40. These chondroblasts proliferate and synthesize collagen fibers and glycosaminoglycans, establishing the extracellular matrix of cartilage. The distribution and orientation of collagen fibers and glycosaminoglycans, along with the degree of chondrocyte calcification, contribute to the multi-layered structure of cartilage. For example, articular cartilage can be broadly categorized into hyaline cartilage and calcified cartilage layers. Cartilage serves various physiological roles, with the most crucial being to provide cushioning and support within joints. It reduces friction between bones and protects them from wear and tear. Moreover, cartilage effectively disperses pressure generated during joint movement, thereby protecting joint tissues. In structures like the nose and ears, the elasticity and flexibility of cartilage allow it to maintain specific shapes 41, 42. Currently, artificially engineered cartilage organoids structures are predominantly composed of uniformly spherical cell clusters or uniformly layered tissues. Research in cartilage organoids mainly focuses on understanding cartilage-related mechanisms, advancing plastic surgery, and promoting cartilage repair (**Figure 3 and Figure 4**).

Cartilage organoids were initially applied to explore cartilage-related mechanisms. In 1990, Somogyi *et al.* pioneered the *in vitro* construction of cartilage organoids. By examining their morphology and ECM, they found that osteoblasts promoted mineralization within cartilage, whereas fibroblasts had inhibitory effects (**Figure 3A**) 43. With the advancement of technology, cartilage organoids have also been utilized to investigate the impact of growth factors (TGF-β) and osmotic pressure on cartilage development (**Figure 3B-C**) 44, 45. Furthermore, cartilage organoids have also been employed to construct organ-on-a-chip models to study inter-tissue interactions. For example, Ertl *et al.* constructed the chondro-synovial organoid chip to simulate cross-talk between individual synovial and cartilage organoids. Co-culturing with synovial organoids, it was demonstrated that cartilage organoids induced a heightened degree of cartilage physiology and structure, along with distinct cellular cytokine responses compared to their respective monocultures, underscoring the significance of inter-tissue cross-talk at the organ level in models of arthritic diseases (**Figure 3D**) 46. In addition to exploring cartilage-related mechanisms, cartilage organoids have also demonstrated remarkable potential in plastic surgery. Notably, one of the most matured applications involves the construction of auricular-shaped cartilage organoids, particularly for reconstructing human ears 47. In 1997, Cao *et al.* constructed the first human-ear mouse with a polyglycolic acid fiber scaffold (**Figure 3E**) 48. Subsequently, Zhou *et al.* advanced the field by constructing human auricular cartilage organoids through co-culturing microtia chondrocytes and bone mesenchymal stem cells (BMSCs) (**Figure 3F**) 49. This approach not only enhanced the shape stability of human auricular cartilage organoids but also effectively reduced construction costs. In 2019, Alsberg *et al.* further advanced the field by enhancing the shape resolution of human auricular cartilage organoids using 3D bioprinting technology (**Figure 3G**) 50. Building on these advances, Lei *et al.* constructed homogeneous and mature human auricular cartilage organoids using synthetically engineered fiber-reinforced SF super elastic absorbent sponges (**Figure 3H**) 51.

In recent years, researchers have increasingly directed their focus towards applying cartilage organoids in cartilage repair. For instance, Lin *et al.* and Yin *et al.* achieved the stacking of cartilage microsphere organoids using materials with self-assembling properties, enabling the construction of larger-volume cartilage organoids (**Figure 4A-B**) 52, 53. Papantoniou *et al.* assembled cartilage microtissues derived from iPSC-derived chondrocytes with callus organoids (COs) sourced from human plasmacytoid dendritic cells to constructed layered osteochondral organoids (**Figure 4C**) 54. It is worth noting that cartilage organoids constructed by Tsumaki *et al.* and Xing *et al.* successfully achieved cartilage repair in primates and canids (**Figure 4D-E**) 55, 56. In addition, Ouyang *et al.* constructed macromass cartilage organoids up to 3 mm in diameter by culturing human polydactyly chondrocytes in customized culture, which can be used as implants to facilitate cartilage defect repair (**Figure 4F**) 57. Based on these studies, it is evident that constructing cartilage organoids requires providing material support for seed cells and directing their chondrogenic differentiation. Consequently, to construct cartilage organoids that closely mimic the natural cartilage structure and function, novel smart biomaterials need to be designed to furnish the chondrogenic microenvironment necessary for seed cells.

## 3. Preparation of silk fibroin-based hydrogel

### 3.1. Characteristics of silk fibroin

**Figure 5:**
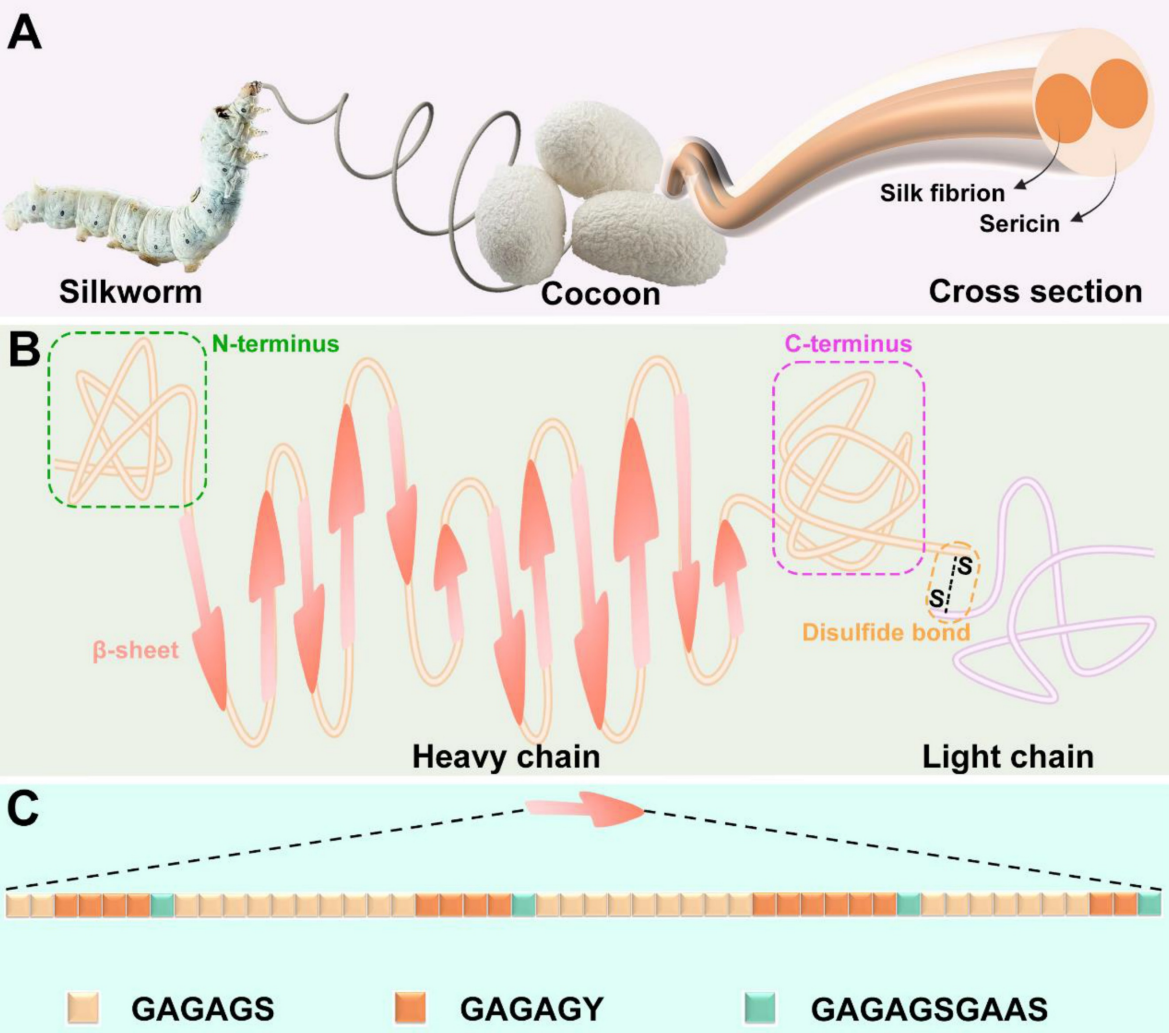
**Source and structure of SF: (A)** SF can be extracted from cocoon of silkworm. **(B)** SF molecule is composed of heavy chain and light chain connected by disulfide bond. **(C)** Crystal structure consists of GAGAGS, GAGAGY and GAGAGSGAAS repetitive sequences. Created with BioRender.com.

SF is a naturally occurring macromolecular material produced by a range of animals, including silkworms, spiders, scorpions, mites, and flies 58. It is worth noting that SF of different origins has obvious differences in structure and properties. Among them, silkworms-derived SF has been widely studied and applied in the clinic because of its unique mechanical properties and abundant yield 59, 60. Hence, this review only discusses the silkworms derived SF. A single silk filament is composed of two strands of SF, enveloped in sericin (**Figure 5A**) 61.

The molecular structure of SF is very complex, which is composed of disulfide-linked heavy chain and light chain (**Figure 5B**). The heavy chain includes non-repetitive C-terminal and N-terminal, along with 11 hydrophilic segments composed of 31 amino acid residues and 12 hydrophobic segments. The hydrophobic segments mainly contain Gly-X repeats, where X can be Ala (65%), Ser (23%), or Tyr (9%). Repeated sequences include Gly-Ala-Gly-Ala-Gly-Ser (GAGAGS), Gly-Ala-Gly-Ala-Gly-Tyr (GAGAGY), and Gly-Ala-Gly-Ala-Gly-Ser-Gly-Ala-Ala-Ser (GAGAGSGAAS). These repetitive sequences can form crystalline β-sheet structures through hydrophobic interactions **(Figure 5C)** 26. Contrary to the heavy chain, the amino acid sequence of the light chain is disordered and tends to form an amorphous structure 62. Recent studies have revealed that SF achieves interfacial self-assembly due to its amphiphilic molecular structure, which promotes the formation of β-sheets at interfaces. This property is particularly useful in hydrogel formation, as the conformation of SF can be modulated by adjusting the water-to-organic phase ratio. These adjustments enable the creation of hydrogels specifically optimized for cartilage regeneration, offering enhanced mechanical properties and bioactivity for improved tissue repair 63.

SF exhibits exceptional physical and chemical properties due to its unique structure and composition. It can adopt four distinct conformations—silk I, silk II, silk III, and an amorphous structure—through intra- and intermolecular interactions. Among these, the amorphous structure and β-sheet-rich silk II conformation endow SF with high mechanical strength and toughness. Furthermore, silk I, silk II, silk III and amorphous structures can be transformed into each other by external effects (temperature, ultrasound, electric field, shear force and pH value) 62. This adaptability makes SF highly suitable for diverse tissue regeneration applications, as its mechanical properties can be finely tuned. As a macromolecular protein material, SF also offers excellent cytocompatibility and biodegradability. It degrades in response to multiple proteases, with its degradation rate primarily controlled by the content of silk II. The degradation products—amino acids and peptides—are non-toxic and can be absorbed by cells, providing essential building blocks for tissue regeneration 76. In addition to these favorable properties, SF's processability allows it to be adapted into various processing methods to meet the complex demands of tissue repair 77. To provide a clearer understanding of SF's properties relative to other biomaterials commonly used in cartilage regeneration, **Table 1** presents a quantitative comparison of SF with collagen, alginate, hyaluronic acid (HA), and Matrigel across key parameters, including mechanical properties, degradation rates, and biological performance.

### 3.2. Cross-linking methods for silk fibroin-based hydrogels preparation

**Figure 6:**
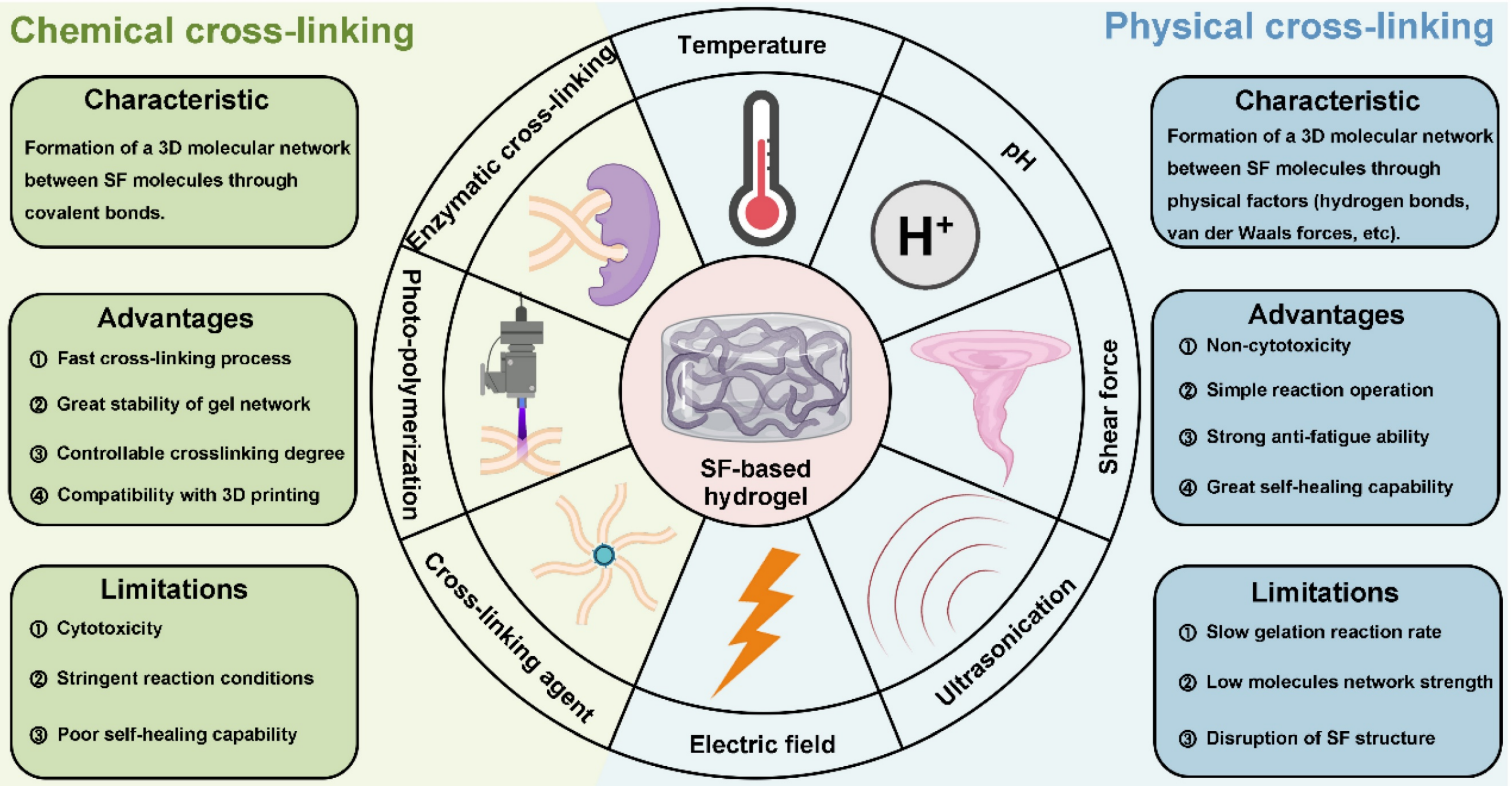
**Cross-linking methods of SF-based hydrogels. Chemical cross-linking: enzymatic cross-linking,** photo-polymerization and cross-linking agent. Physical cross-linking: temperature, pH, shear force ultrasonication and electric field. Created with BioRender.com.

**Figure 7:**
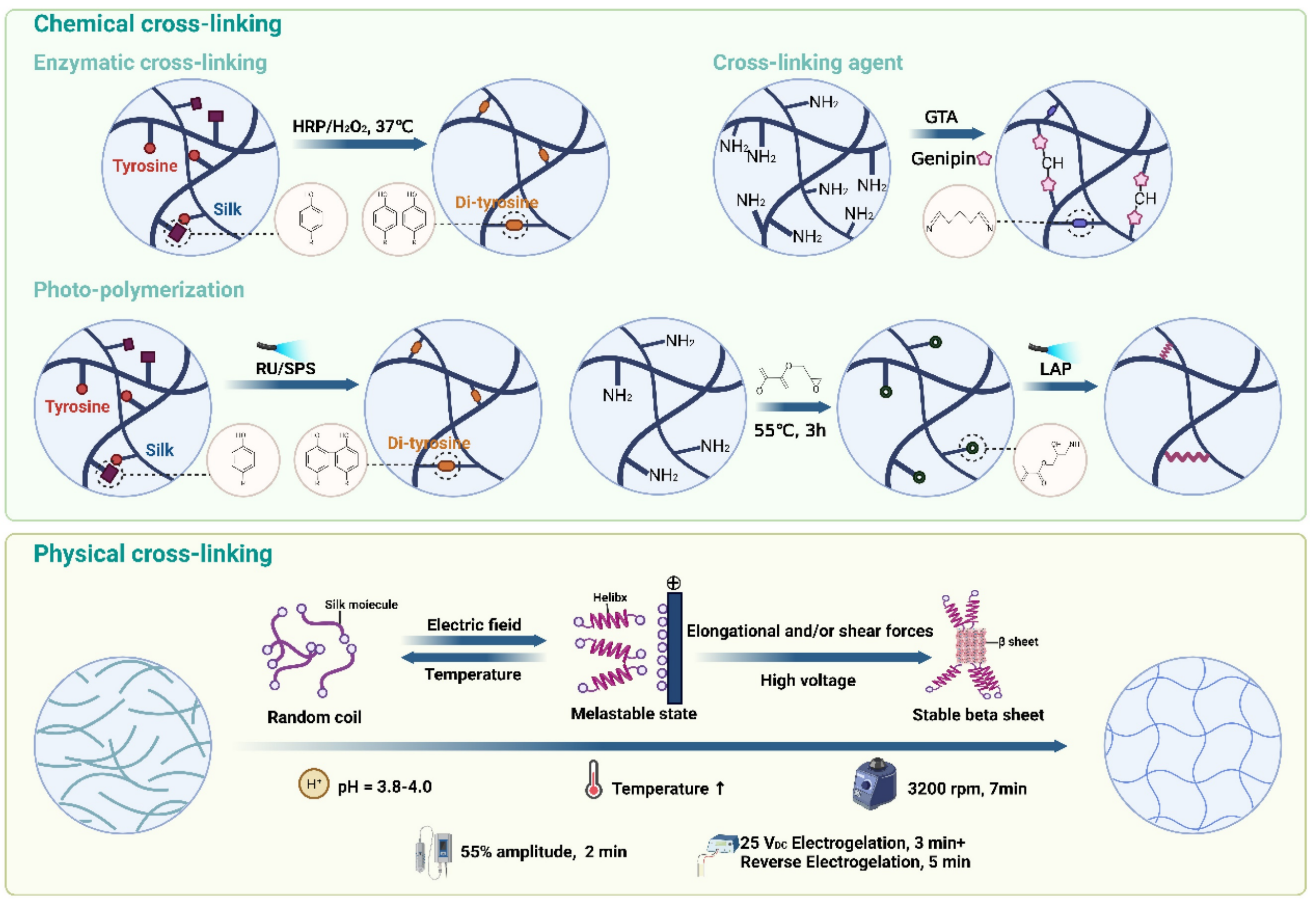
**Schematic representation of the mechanisms and preparation methods of different cross-linking approaches for SF-based hydrogels.** Created with BioRender.com.

As previously mentioned, SF can be processed using various methods, including hydrogel preparation through cross-linking. Cross-linking methods are generally categorized into chemical and physical approaches 78. Chemical cross-linking promotes the formation of covalent bonds by adding enzymes, cross-linking agents and photo-initiators to accelerate SF gelation. In contrast, physical cross-linking involves the self-assembly of SF into hydrogels by regulating physical parameters such as temperature, pH, shear force, ultrasound, and electric fields, each method offering distinct advantages and limitations (**Figure 6, Table 2**). The schematic in **Figure 7** provides an overview of the mechanisms and preparation techniques employed in these cross-linking strategies for SF hydrogels.

#### 3.2.1. Chemical cross-linking

##### Enzymatic cross-linking

In recent years, enzyme cross-linked hydrogels have attracted wide attention in the biomedicine field. For SF-based hydrogels, enzymes facilitate the formation of intermolecular covalent bonds by activating functional groups within SF. Additionally, enzymatic cross-linking induces the formation of an ECM-like elastic structure by controlling β-sheet formation, resulting in hydrogels with stable structures, controllable mechanical properties, and non-toxic effects on cells 79. This method also supports cell encapsulation due to its cross-linking process being conducted at physiological pH and temperature 80.

Among the numerous enzymatic cross-linking reactions, horseradish peroxidase (HRP) mediated enzymatic cross-linking reaction is the most commonly used 81, 82. It has the advantages of high selectivity, mild reaction conditions and no toxic components 83, 84. HRP is typically combined with H2O2 to induce SF cross-linking by oxidizing tyrosine residues into o-quinone residues. These o-quinone residues then react with phenol or aniline to form covalent bonds, leading to intermolecular or intramolecular cross-linking 85. For example, Hasturk *et al.* prepared SF/tyramine-substituted SF (SF-TA) composite hydrogel by using HRP and H_2_O_2_ 86. The composite hydrogel exhibited adjustable mechanical properties, degradability, and excellent cytocompatibility, making it promising for cartilage defect repair due to its cell encapsulation capability. Li *et al.* further developed an SF-gelatin (SF-GT) hydrogel with a macroporous structure using HRP/H2O2 in combination with 3D bioprinting 87. SF-GT hydrogel had structural stability, mechanical properties and adjustable degradation rate for cartilage reconstruction. Additionally, SF-GT hydrogel could induce stem cells to synthesize Col II at a higher level and show hyaline cartilage phenotype.

##### Photo-polymerization

Photo-polymerization is a widely used chemical cross-linking method that utilizes a photo-initiator and light (ultraviolet, visible, or gamma rays) to control the cross-linking process 88. During photo-polymerization, the photo-initiator absorbs light energy and cleaves to produce free radicals, which subsequently react with unsaturated bonds in SF to induce cross-linking. The primary advantage of photo-polymerization is its extremely rapid cross-linking rate 89, 90. For instance, Cui *et al.* successfully cross-linked SF within 1 minute using tris(2,2-bipyridyl)dichlororuthenium(II) hexahydrate and sodium persulfate as photo-initiators 91. The SF-based hydrogels had stable mechanical properties and supported the long-term culture of human articular chondrocytes and cartilage tissue regeneration. In addition, there is a special photo-polymerization method using high-intensity gamma-ray without adding photo-initiators. This method can completely remove the toxic effects caused by photo-initiators residues 92. For example, Kim *et al.* prepared chemically cross-linked SF hydrogels by using Co-60 derived gamma-ray (SF C-gel) 93. They found that SF C-gel was biocompatible and could promote the attachment and proliferation of hMSCs.

##### Cross-linking agents

Cross-linking agent molecules can accelerate SF cross-linking by reacting with reactive groups such as -OH, -NH_2_ and -COOH in SF 94. Compared to enzymes and photo-initiators, cross-linking agents are more cost-effective and can improve the mechanical properties of hydrogels 59. Glutaraldehyde (GTA) is the most widely used cross-linking agent, which can promote SF cross-linking by reacting with the phenolic group of tyrosine. For instance, Srisawasdi *et al.* prepared polycarbazole/SF (SF/PCZ) hydrogels with glutaraldehyde as cross-linking agent 95. They found that SF/PCZ hydrogel had good dielectric properties and excellent toughness. However, the biotoxicity of GTA limits its applications in tissue engineering and medicine. In contrast, genipin is a promising natural small molecule cross-linking agent due to its excellent biocompatibility. Considering this, Min *et al.* designed chitosan/SF hydrogels loaded with kartogenin (KGN) and platelet-derived growth factor BB (PDGF-BB) by using genipin as cross-linking agent 96. The hydrogels allowed for the sustained release of KGN and PDGF-BB, supporting the growth of seed chondrocytes and maintaining their phenotype, demonstrating potential in cartilage tissue engineering.

#### 3.2.2. Physical cross-linking

##### Temperature

Temperature significantly affects the cross-linking of proteins, including SF 97. Increasing the temperature can promote SF cross-linking by enhancing the Brownian motion of SF molecules and increasing the effective collision rate between them. Additionally, elevated temperatures can disrupt the free energy state of SF molecules, exposing internal hydrophobic regions and facilitating the transition from random coil to β-sheet structures, thereby enhancing hydrophobic interactions and accelerating cross-linking 98. For instance, Kim *et al.* researched the effect of cross-linking temperature for SF hydrogels 99. They found that the cross-linking rate and compressive modulus of SF hydrogels increased with increasing cross-linking temperature within a certain range.

##### pH

In addition to temperature, the pH of the SF solution is also a critical factor in SF cross-linking. When the pH of the solution approaches the isoelectric point of SF (pH = 3.8-4.0), the electrostatic repulsion between SF molecules is minimized, making the molecules more prone to aggregation and cross-linking 94. In this condition, the SF molecules are unstable and prone to aggregation and cross-linking. Therefore, adjusting the pH value of the solution is an effective method to induce SF cross-linking. For example, Nagarkar *et al.* investigated SF cross-linking by changing pH via adding HCl 100. They found that adjusting the solution pH from 8 to 2 could prepare weak SF hydrogels. Additionally, Fini *et al.* designed and developed SF-based hydrogel though regulating pH via adding citric acid to SF solution 101. They found that the hydrogel had good mechanical properties and excellent cytocompatibility. Additionally, *in vivo* experiments demonstrated that the hydrogel showed non-inflammatory response after implantation and stimulated cells to produce TGF-β1 to induce tissue regeneration.

##### Shear force

The shear force cross-linking method typically involves high-speed vortex treatment on SF solution 102. High-speed vortex treatment accelerates β-sheet generation by stretching SF molecules and changing their orientation to promote SF cross-linking 103. Moreover, this method can also be used to prepare SF-based hydrogels with directional structures. For example, Chen *et al.* fabricated SF/sodium surfactin hydrogels with directional three-dimensional structure by vortex treatment 104. The hydrogel could accelerate 3D directed tissue regeneration due to its excellent anisotropy. Moreover, Kasoju *et al.* fabricated SF hydrogel with good mechanical properties by combining methanol treatment and vortex treatment 105. These hydrogels demonstrated effective cell encapsulation and controlled drug release capabilities.

##### Ultrasonication

Ultrasonication is a physical cross-linking method commonly used to prepare SF hydrogels. The effect of this method is similar to the anterior silk gland of silkworm, which promotes SF cross-linking through local temperature increase and shear force 106. This method is highly stable and controllable, as it allows for adjustments in output energy and duration. Importantly, it effectively avoids the toxicity issues associated with additives like photo-initiators and cross-linking agents 107. For example, Byram *et al.* designed and developed SF/xanthan gum hydrogels by ultrasonication 108. Additionally, due to their cartilage ECM-like microstructure, these hydrogels showed potential for applications in cartilage tissue engineering.

##### Electric field

As we mentioned before, SF is negatively charged in neutral solutions due to its isoelectric point (pH=3.8-4.0). Under an electric field, SF molecules aggregate near the anode, forming micelles that subsequently assemble into hydrogels through physical entanglement of molecular chains 109. For instance, Liu *et al.* constructed SF electrogels with excellent mechanical properties via a low-voltage electric field 110. They found that the hydrogel had excellent biocompatibility and drug loading capacity. In addition, gradient structure hydrogels which are more suitable for cartilage repair can be prepared by the electric field. Consider this, Xu *et al.* developed multi-functional beta-sheet rich silk nanofibers (BSNF) hydrogels with adjustable gradient mechanical strength and structure in electric field 111. BSNF hydrogel could regulate BMSCs to differentiate into chondrocytes to promote cartilage repair due to its gradients structure.

### 3.3. Functional modifications

As we mentioned in *3.1. Characteristics of silk fibroin*, SF has excellent biocompatibility, biodegradability and mechanical properties. However, after various physical and chemical treatments, the molecular structure of SF is destroyed, which leads to the unsatisfactory mechanical strength of pure SF hydrogel. In addition, pure SF hydrogel has certain disadvantages, such as insufficient water retention, poor antibacterial properties and unsatisfactory cartilage repair properties 138. In order to improve SF hydrogel, it is an effective strategy to prepare composite SF hydrogel by mixing some functional materials into SF solution 139. These functional materials can be divided into synthetic materials and natural materials (**Table 3**).

#### 3.3.1. Synthetic material modification

Graphene oxide is a kind of functional carbon allotrope. Recently, it has attracted increasing attention in the field of materials due to its excellent mechanical strength, attractive surface volume ratio, high water solubility, easy solution processing and chemical functionality 140. The incorporation of GO into SF solutions can significantly enhance the mechanical properties of SF-based hydrogels. For example, Wang *et al.* developed nano-hydroxyapatite-GO/SF hydrogels using click chemistry, resulting in enhanced mechanical strength compared to pure SF hydrogels, with a compressive modulus of 95.4 ± 2.0 kPa 112. Furthermore, the addition of GO can improve the toughness of SF hydrogels. Balu *et al.* fabricated regenerated SF (RSF)/GO nanocomposite hydrogels using RuBPY as a photo-initiator, achieving mechanical properties superior to natural cartilage, with a Young's modulus of 8 MPa and tensile toughness of 2.4 MJ/m^3^ 113.

Synthetic polymers are also commonly used to prepare composite hydrogels. Among them, polyethylene glycol (PEG) has received wide attention due to its versatility in molecular weight, topology (linear, branched, star-shaped) 139, 141. Interestingly, the incorporation of PEG can endow the SF composite hydrogel with the injectable property. For example, Zhang *et al.* prepared injectable BMSC-encapsulated dual-network SF-PEG composite hydrogels via ultrasonication, resulting in hydrogels with a high cross-linking rate, excellent biocompatibility, and strong mechanical strength. Additionally, these dual-network SF-PEG hydrogels promoted cartilage repair by enhancing BMSC chondrogenic differentiation 114. Furthermore, derivatives of PEG can also strengthen the cartilage repair ability of SF hydrogels. For instance, Achachelouei *et al.* fabricated SF/poly(ethylene glycol) dimethacrylate (PEGDMA) hydrogels with adjustable mechanical properties by photo-polymerization 115. They found that the compression modulus of SF/PEGDMA hydrogel was related to the ratio of SF to PEGDMA. Meanwhile, Bandyopadhyay *et al.* constructed silk methacrylate (SilMA)-PEG diacrylate hydrogel with excellent mechanical properties and adjustable degradability via photo-polymerization 116. Importantly, this hydrogel was suitable for chondrocytes-laden 3D biological printing to accelerate cartilage repair.

Apart from PEG, the performance of SF hydrogels can be enhanced by mixing with poly(N-vinylcaprolactam), poly vinyl alcohol, poloxamer 142. For instance, Whittaker *et al.* constructed RSF-poly(N-vinylcaprolactam) double network (DN) hydrogel by a rapid one-pot method 117. Compared with SF hydrogels, poly(N-vinylcaprolactam) enhanced elasticity and toughness of hybrid hydrogels. Furthermore, Subramanian *et al.* developed Mo_3_Se_3_-PVA-SF nanowire hydrogel with remarkable mechanical properties by using glutaraldehyde as cross-linking agent 118. They found the composite could stimulate the expression of collagen to accelerate tissue repair. It is worth noting that POL can impart injectability to SF hydrogel 120. For example, Min *et al.* constructed an injectable alginate-poloxamer (ALG-POL)/SF hydrogel though using HRP and H_2_O_2_ 119. The injectability of this hydrogel was attributed to its solution-gel transition properties at physiological temperature. In addition, ALG-POL/SF hydrogel accelerated cartilage regeneration by promoting chondrocyte proliferation.

MXenes, as novel 2D nanomaterials composed of transition metal complexes, had been applied in biomedicine due to their metallic conductivity, piezoelectricity, excellent hydrophilicity, and diverse surface chemical properties. Jiang *et al.* utilized enzyme crosslinking to combine MXene nanosheets with SF, forming a piezoresistive nanocomposite hydrogel that facilitated bone tissue regeneration by restoring the electrical microenvironment 121. Additionally, Yang *et al.* developed an injectable SF/MXene conductive hydrogel developed through ultrasound techniques, which acted as a stem cell carrier and enabled *in vivo* electrical stimulation for repairing brain tissue damage 122.

#### 3.3.2. Natural material modification

The mixing of synthetic materials in SF hydrogels may introduce cytotoxicity. Conversely, the combination of SF with natural materials can avoid the toxicity problems associated with synthetic materials 143. Currently, composite hydrogels have been prepared by combining with GT, collagen, cellulose or HA 144, 145.

Collagen (Col) is the most abundant natural polymer in ECM with remarkable biocompatibility, negligible immunogenicity and strong biological activity. Notably, collagen can enhance cartilage regeneration effects as it promotes the attachment and chondrogenic differentiation of MSCs 123. For example, Zhang *et al.* developed an injectable BMSC-laden collagen-PEG/SF DN hydrogel via ultrasonication, which exhibited enhanced mechanical strength and cytocompatibility, ultimately accelerating cartilage regeneration 124.

GT is a mixture of large polypeptides, denatured from collagen. GT is a promising material in tissue engineering attributed to its high swelling and thermal inversion properties 125. In addition, GT can form an interpenetrating network (IPN) with SF to enhance the compressive moduli of SF-based hydrogel. Park *et al.* fabricated GT-SF IPN hydrogels through microbial transglutaminase-induced cross-linking, resulting in biodegradable, non-cytotoxic hydrogels with superior mechanical properties compared to individual GT or SF hydrogels. The resulting composite hydrogel also promoted cell adhesion and proliferation 126.

Cellulose is a polysaccharide biomolecular material available in various sources 146-148. Noticeably, cellulose can be modified in several ways to generate cellulose derivatives 149. These cellulose derivatives strengthen the compressive modulus of SF-based hydrogels by inducing the generation of β-sheets. For instance, Luo *et al.* fabricated RSF/hydroxypropyl methyl cellulose (HPMC) hydrogel with excellent mechanical properties 127. The maximum compressive and tensile modulus of this hydrogel exceeded 1.0 MPa.

Chitosan (CS) is a kind of polysaccharide biopolymer, which has an inherent linear structure, excellent biocompatibility and environmental responsiveness. It has been proved that SF hydrogels can be endowed with environmental responsiveness by combining with CS 128. For example, Xu *et al.* developed pH responsiveness SF/CS hydrogel by chemical cross-linking 129. Meanwhile, Yu *et al.* prepared CS/SF/amino-functionalized mesoporous silica hydrogel through using genipin as cross-linking agent 130. They found that the excellent injectability of the composite hydrogel was due to its sensitive thermal responsiveness at physiological pH. Additionally, CS-SF composite hydrogels can achieve controlled drug release. Dong *et al.* prepared a p-hydroxybenzene propanoic acid-modified chitosan (PC)/SF hydrogel using an HRP-mediated enzymatic cross-linking reaction, which sequentially released bioactive molecules to induce MSC homing and chondrogenic differentiation 131.

HA is a common biodegradable, non-immunogenic and non-inflammatory polysaccharide in the human body. It has been reported that mixing HA can enhance the storage modulus and compressive modulus of SF hydrogels 132, 150. For example, Ziadlou *et al.* designed and developed HA-TA /SF composite hydrogels via enzymatic cross-linking 133. Compared with pure SF hydrogel, they found this hybrid hydrogel had enhanced storage modulus and drug releasing ability to stimulate cartilage regeneration, remarkable mechanical properties and drug release ability. Moreover, Wang *et al.* also fabricated an SF-HA composite hydrogel scaffold by combining SF/HA-Tyr hydrogel with SF sponge, finding that this biomaterial accelerated cartilage repair due to its excellent mechanical properties and MSC recruitment ability 134. Fan *et al.* compounded SF with aldehyde-HA to create a dynamic network that delayed or interrupted the β-sheet-induced hardening of SF chains, producing a hydrogel matrix with mechanical properties similar to biological tissues 135.

Glycyrrhizic acid (GA), a natural compound from licorice roots, effectively inhibits intrinsic inflammation and, when combined with SF, modifies the mechanical properties of materials to reduce mechanical stress and lower friction coefficients *in vivo* after implantation. Zhang *et al.* developed a nanocomposite hydrogel for spinal cord repair by creating an interpenetrating polymer network with self-assembled GA as the first network and photo-crosslinked SilMA as the second network 136.

Deoxyribonucleic acid (DNA) is a natural macromolecular material, and hydrogels prepared using DNA exhibit unique programmability, good injectability, controllable mechanical properties, and are adaptable for 3D cell printing 17. It has been demonstrated that DNA hydrogels have significant potential in protecting seed cells, particularly in the field of cartilage repair 151. Recently, our team introduced DNA into SF hydrogels, imparting them with controllable surface rigidity to regulate the chondrogenic differentiation of BMSCs, thereby achieving a breakthrough in cartilage repair 137. Details will be discussed in *4.3. Acceptable mechanical properties*.

## 4. Advantages of silk fibroin-based hydrogels in the construction of cartilage organoids

**Figure 8:**
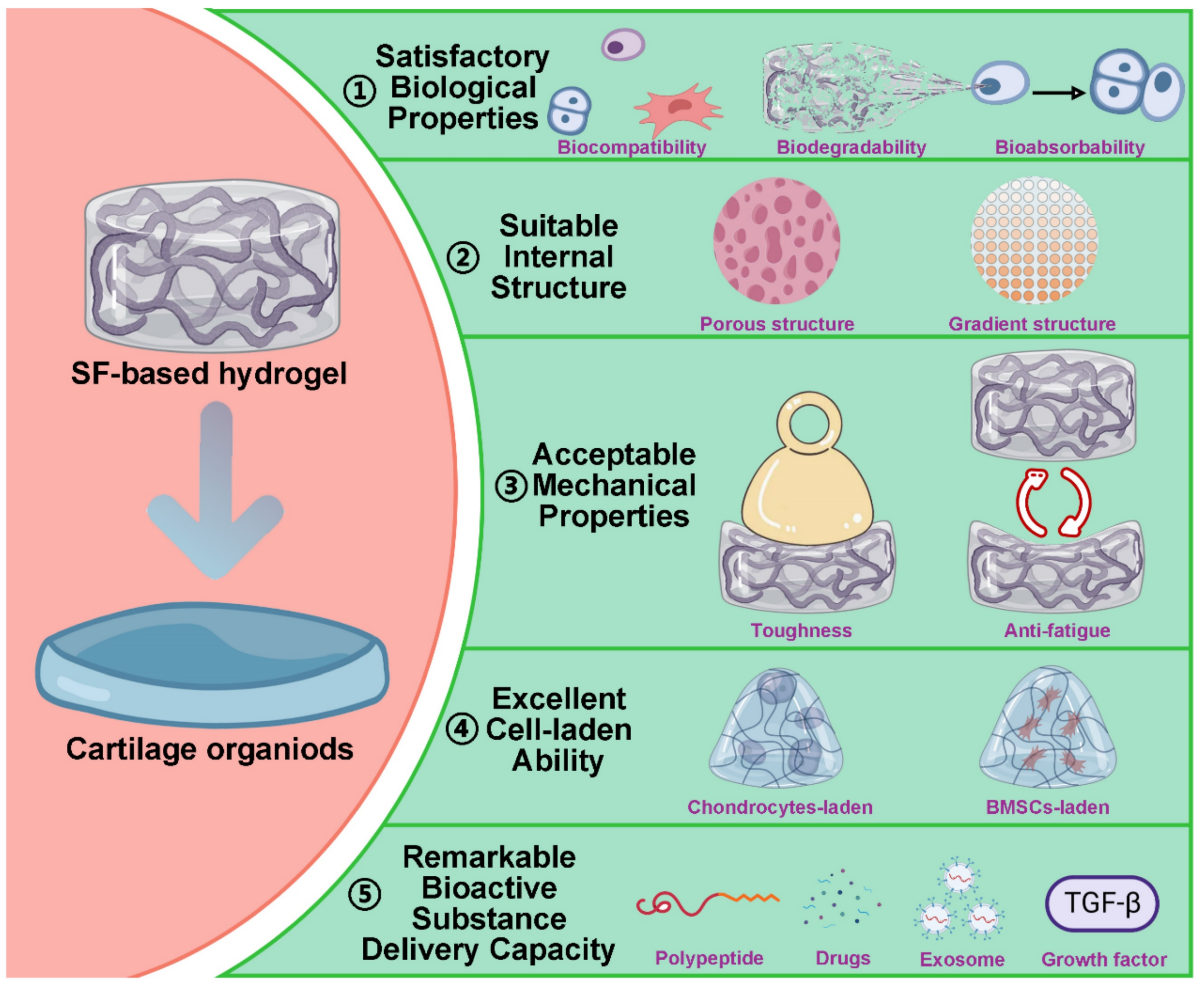
** Advantages of SF-based hydrogels in the construction of cartilage organoids:** satisfactory biological properties, suitable internal structure, acceptable mechanical properties, excellent cell-laden ability and remarkable bioactive substance delivery capacity. Created with BioRender.com.

Based on multiple cross-linking and modifications strategy, SF-based hydrogels have great potential in promoting cartilage regeneration 152. Meanwhile, it also demonstrates that SF-based hydrogels have certain advantages in constructing cartilage organoids, including: satisfactory biological properties, suitable internal structure, acceptable mechanical properties, excellent cell-laden ability and remarkable bioactive substance delivery capacity (**Figure 8**).

### 4.1. Satisfactory biological properties

An ideal hydrogel for constructing cartilage organoids is inadmissible to inhibit the normal metabolic activity of cells, cause inflammatory responses, or induce apoptosis. For pure SF hydrogels, the problem of immunogenicity can be avoided by degumming completely. For SF-based composite hydrogels, the additional materials should have excellent biocompatibility and biodegradability. In addition, the construction of cartilage organoids is a dynamic and continuous process. Therefore, the SF hydrogel applied to the construction of cartilage organoids should have an appropriate degradation rate 153. During the construction of cartilage organoids, SF hydrogels should be degraded gradually to reserve space for newborn cartilage tissue. Furthermore, all the degradation products of SF hydrogel are non-cytotoxic and can be absorbed by cells to provide a nutritional basis for cartilage regeneration 65.

### 4.2. Suitable internal structure

SF-based hydrogels can mimic the ECM of cartilage due to their hydrophilic three-dimensional porous structure 91. As a substitute for ECM, the porous structure of SF-based hydrogel can provide channels for the diffusion of nutrients into chondrocytes, the migration of chondrocytes and the removal of metabolic waste 87, 154, 155. It is worth noting that the extracellular matrix of cartilage is heterogeneous. From outside to inside, cartilage tissue can be divided into superficial zone, middle zone, deep zone and calcified zone. The composition and content of ECM are different in each zone 156. Therefore, the ideal SF hydrogel for constructing cartilage organoids should have a multi-level or a gradient structure. For instance, Guo *et al.* fabricated SF/R5 peptide composite hydrogel with gradient structure 157. The hydrogel could regulate the differentiation of BMSCs and accelerate cartilage repair.

### 4.3. Acceptable mechanical properties

Cartilage, a smooth, flexible, and durable tissue, plays a supportive and protective role within the skeletal system. Therefore, the construction of cartilage organs requires biomaterials to provide sufficient mechanical support. As we mentioned in *3.3. Functional modification*, mixing other materials can improve the mechanical strength of SF-based hydrogel. It is worth noting that the cartilage is in a constant cycle of compression and rebound. Meanwhile, during cartilage regeneration, chondrocytes are more active in a dynamic environment 158, 159. Hence, it is preferable to construct cartilage organoids in a dynamic environment, which requires SF hydrogels with excellent anti-fatigue ability. Consider this, Huang *et al.* prepared host-guest interactions SF hydrogel (HG-SF hydrogel) with high remarkable anti-fatigue ability by modifying SF with cholesterol or β-cyclodextrin 160. The hydrogel could remain in its original state after 10 times 60% stress compression without any deformation or strength decrease. Interestingly, HG-SF hydrogel could repair itself after stress injury, which showed that it had great potential to construct cartilage organoids in a dynamic environment. Additionally, our recent work found that the introduction of DNA supramolecules effectively aggregated SF molecules, inducing the formation of β-sheet structures in SF and consequently adjusting the surface rigidity of the DNA-SF hydrogels. Simultaneously, we observed that DNA-SF hydrogels with a moderate surface rigidity could induce the secretion of collagen-containing ECM by upregulating the TGF-β and Wnt signaling pathways, thereby promoting the chondrogenic differentiation of BMSCs. *In vivo* experimental results also demonstrated that DNA-SF hydrogels with moderate surface rigidity could expedite the cartilage repair process 137. Based on this, Su *et al.* integrated photo-crosslinking with self-assembly technology to develop a novel DNA-SF hydrogel microsphere using a microfluidic system. This microsphere material offers a new option for constructing and sustaining long-term culture of cartilage organoids 161.

### 4.4. Excellent cell-laden ability

**Figure 9:**
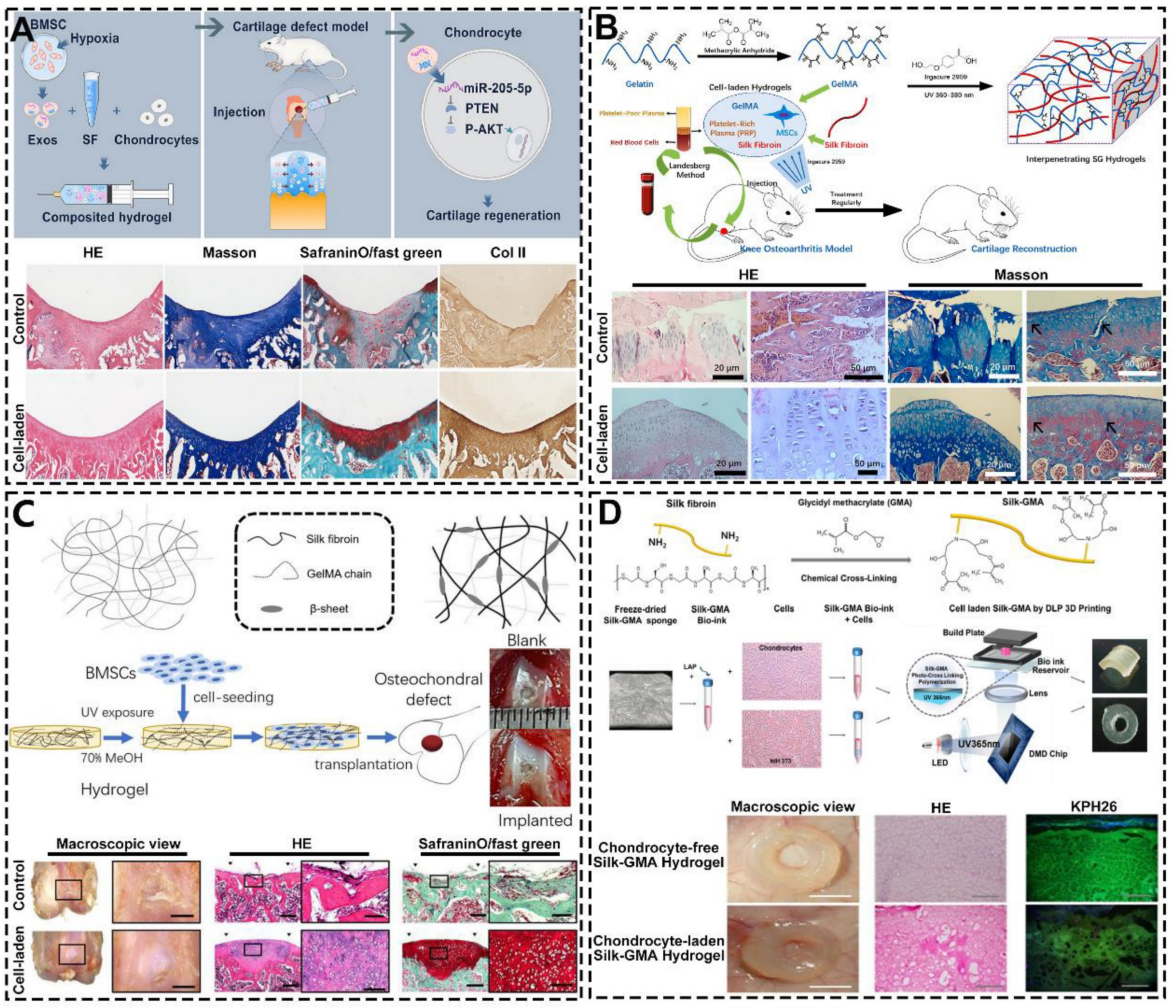
**Excellent cell-laden ability of SF-based hydrogels: (A)** Injectable chondrocytes-laden SF-based hydrogel promoted cartilage regeneration via the miR-205-5p/PTEN/AKT pathway; Reproduced with permission from ref 163; Copyright 2022, Elsevier. **(B)** MSCs-laden GelMA/SF hydrogel with the incorporation of PRP for treating osteoarthritis to reconstruct cartilage; Reproduced with permission from ref 166; Copyright 2023, Elsevier. **(C)** BMSCs-laden GelMA-SF IPN hydrogel for osteochondral defect repair; Reproduced with permission from ref 167; Copyright 2023, MDPI; **(D)** Digital light processing 3D printed chondrocyte-laden Silk-GMA hydrogel for trachea cartilage tissue engineering; Reproduced with permission from ref 168; Copyright 2019, Elsevier.

Based on its structure and chemical composition, SF-based hydrogel has excellent cell-laden ability. In the field of constructing cartilage organoids, SF-based hydrogel with excellent cell-laden ability can load chondrocytes and promote their growth and proliferation to achieve cartilage regeneration 162. For instance, Shen *et al.* prepared injectable SF hydrogel containing articular chondrocytes and hypoxia preconditioned exosomes by ultrasonication (**Figure 9A**) 163. They found this hydrogel significantly enhances the expression of cartilage marker genes in ACs. Additionally, histological analysis showed that the surface of the cartilage defect area repaired by cell-laden hydrogel was smooth and the regenerated cartilage arranged regularly. However, chondrocyte-laden SF hydrogels are difficult to rapidly construct cartilage organoids due to the poor mitotic activity of chondrocytes. To solve this problem, SF hydrogels can be loaded with stem cells that have the high proliferative capacity and chondrocyte differentiation potential 164, 165. For example, Chen *et al.* designed and synthesized MSCs-laden GT methacrylate/SF (GelMA/SF) hydrogel with loading platelet-rich plasma (PRP) (**Figure 9B**) 166. As shown by HE and Masson staining, after the intervention of MSCs-laden GelMA/SF hydrogel, the surface of articular cartilage was smooth, the chondrocytes were arranged neatly. Meanwhile, Zheng *et al.* constructed BMSCs-laden interpenetrating network GelMA/SF composite hydrogel through UV photo-polymerization (**Figure 9C**) 167. The macroscopic results, HE and SafraninO/fast green staining showed that osteochondral repair effect of cell-laden group was significantly superior than others group. It is worth noting that the hydrogel can also match 3D biological printing technology to adapt to the regeneration and repair of a variety of cartilage tissues. For instance, Hong *et al.* fabricated chondrocyte-laden SF-glycidyl-methacrylate (Silk-GMA) hydrogel by using 3D bioprinting technology (**Figure 9D**) 168. The *in vivo* experiments showed that the characteristics of the new cartilage tissue (cell morphology, proteoglycan and collagen distribution) of the chondrocyte-laden hydrogel group were closer to the natural cartilage than the chondrocyte-free hydrogel group.

### 4.5. Remarkable bioactive substance delivery capacity

**Figure 10:**
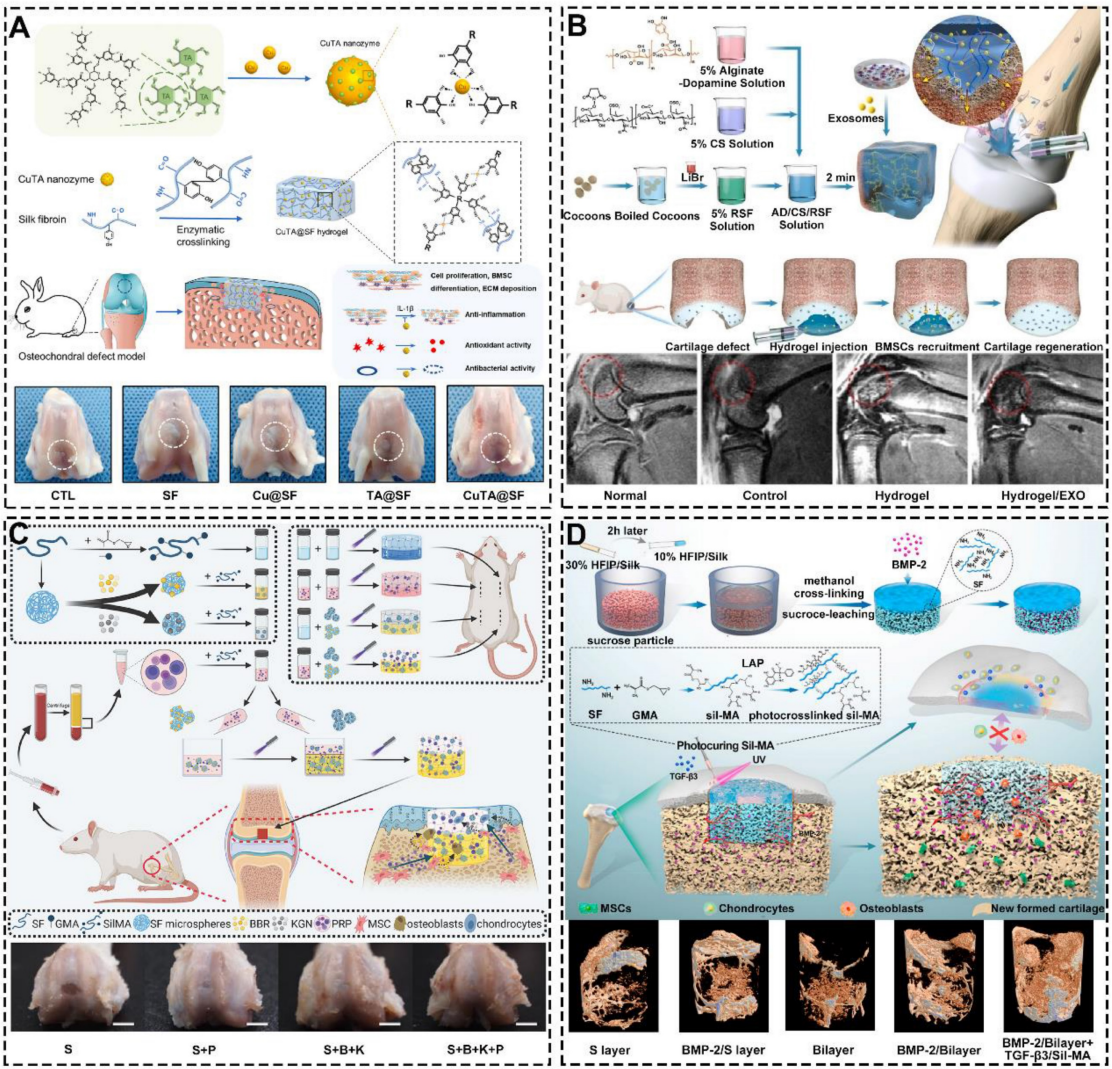
**Remarkable bioactive substance delivery capacity of SF-based hydrogels: (A)** Silk-based hydrogel incorporated with CuTA nanozymes for enhanced osteochondral defects regeneration via providing a suitable microenvironment; Reproduced with permission from ref 176; Copyright 2022, Elsevier. **(B)** Injectable SF-based hydrogel with loading exosomes promoted endogenous cell recruitment and cartilage defect regeneration; Reproduced with permission from ref 180; Copyright 2021, Elsevier. **(C)** SilMA bilayer hydrogel with loading BBR, KGN and PRP stimulated osteochondral defects repair; Reproduced with permission from ref 181; Copyright 2022, Elsevier; **(D)** Photocurable SF-based hydrogel with loading TGF-β3 accelerated cartilage integration; Reproduced with permission from ref 182; Copyright 2021, Elsevier.

During natural organogenesis (from cells to organs), cells need to undergo multiple differentiations in different directions to form multiple cell populations that reassemble into functionally intact organs 169-172. The construction of cartilage organoids parallels this process, requiring the directed induction of cells. Due to its excellent bioactive substance delivery capability, SF-based hydrogels can regulate cell proliferation, migration, and differentiation by loading polypeptides, drugs, exosomes, or growth factors 173-175. For instance, Cao *et al.* developed a multifunctional silk-based hydrogel incorporated with metal-organic framework nanozymes (**Figure 10A**) 176. This hydrogel had the synergistic effects of antioxidation, anti-inflammation and antibacterial, which could regulate the fate of cells and accelerate the regeneration of osteochondral defects. In addition, SF hydrogels loaded with exosomes can mimic the induction of cartilage regeneration by primary cells with no immune rejection 177-179. For example, Zhang *et al.* fabricated alginate-dopamine/chondroitin sulfate/regenerated SF/exosome (AD/CS/RSF/EXO) hydrogel by enzymatic cross-linking (**Figure 10B**) 180. The hydrogel had good injectability, mechanical strength and stickiness. Besides, could induce homing and chondrogenic differentiation of BMSCs. Notably, SF hydrogels loaded with growth factors regulates the migration behavior of cells. For instance, Jiang *et al.* prepared bilayer SilMA hydrogel loaded with Platelet-rich plasma (PRP), SF-KGN microspheres and SF-berberine (BBR) microspheres through stratified photocuring (**Figure 10C**) 181. PRP enhanced the hydrogel's effect on BMSC migration and pre-differentiation, while the layered incorporation of SF-KGN and SF-BBR microspheres enabled long-term regulation of BMSC differentiation and cartilage regeneration. Meanwhile, Wu *et al.* constructed silk-GMA (Sil-MA) hydrogel with loading TGF-β3 through UV photo-polymerization (**Figure 10D**) 182. They found that the hydrogel could promote the migration and chondrogenic differentiation of BMSCs. Importantly, the hydrogel can act as a bridge to induce integration of newborn cartilage and native cartilage. Additionally, Xia *et al.* utilized click chemistry to coat SF microspheres with heparin disaccharide. They then used heparin disaccharide for selective adsorption of IL-4, while LOX pDNA was preloaded onto the SF microspheres through electrostatic interactions. These functionalized SF microspheres were designed to be injected into the cartilage cavity, where they promote macrophage M2 polarization, with LOX facilitating collagen crosslinking to aid in cartilage repair 183.

## 5. Silk fibroin-based hydrogels in iterative optimization for cartilage organoids

**Figure 11:**
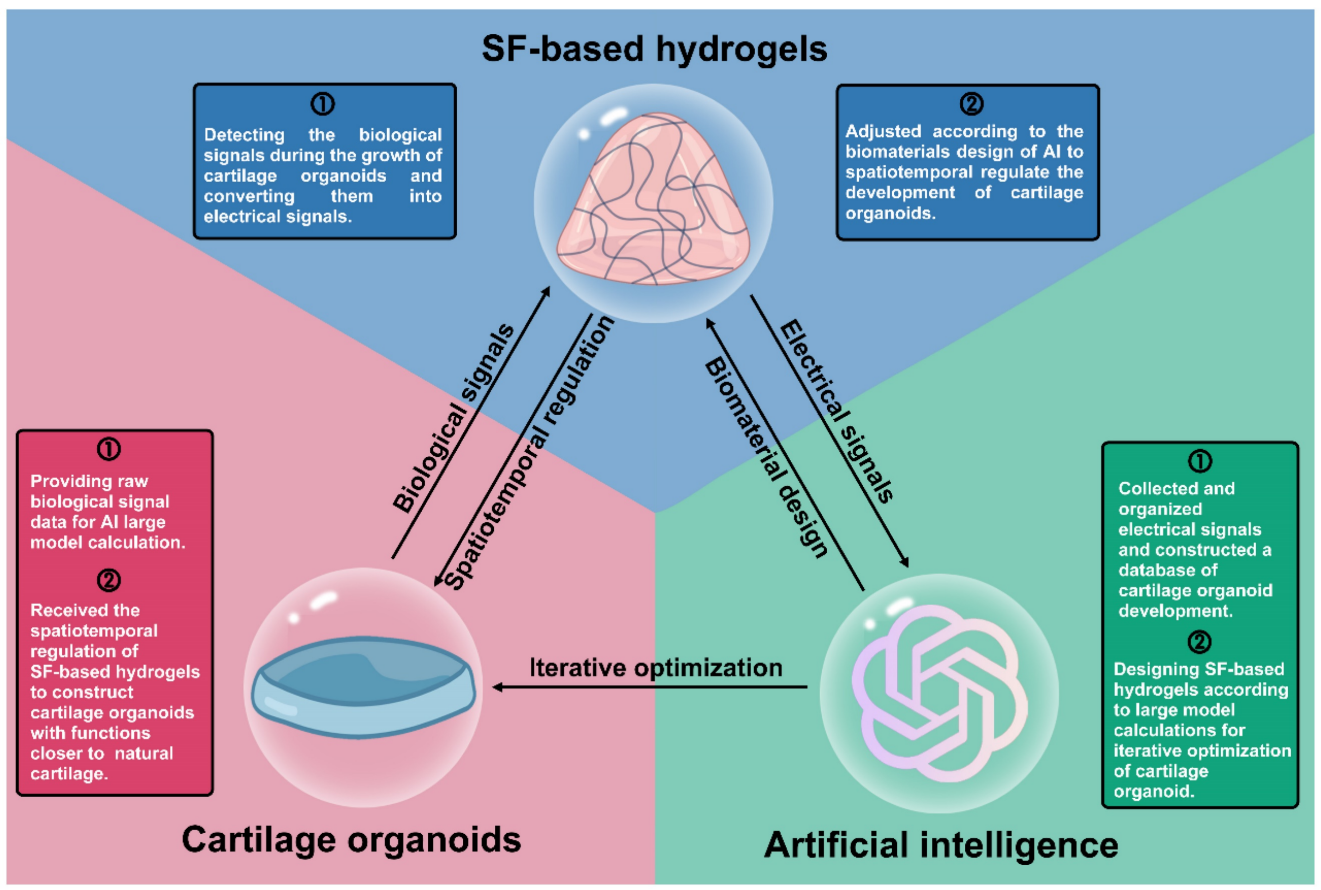
Schematic diagram of triangular relationship between cartilage organoids, SF-based hydrogels and artificial intelligence.

The construction of cartilage organoids is not a one-time process but requires continuous iterative optimization. To meet these needs, SF-based hydrogels must also be continuously upgraded. AI, regarded as the "design master" in life sciences, can utilize large-scale computational models to optimize the design of SF-based hydrogels, thereby enhancing their performance for cartilage organoid construction (**Figure 11**) 184.

**Figure 12:**
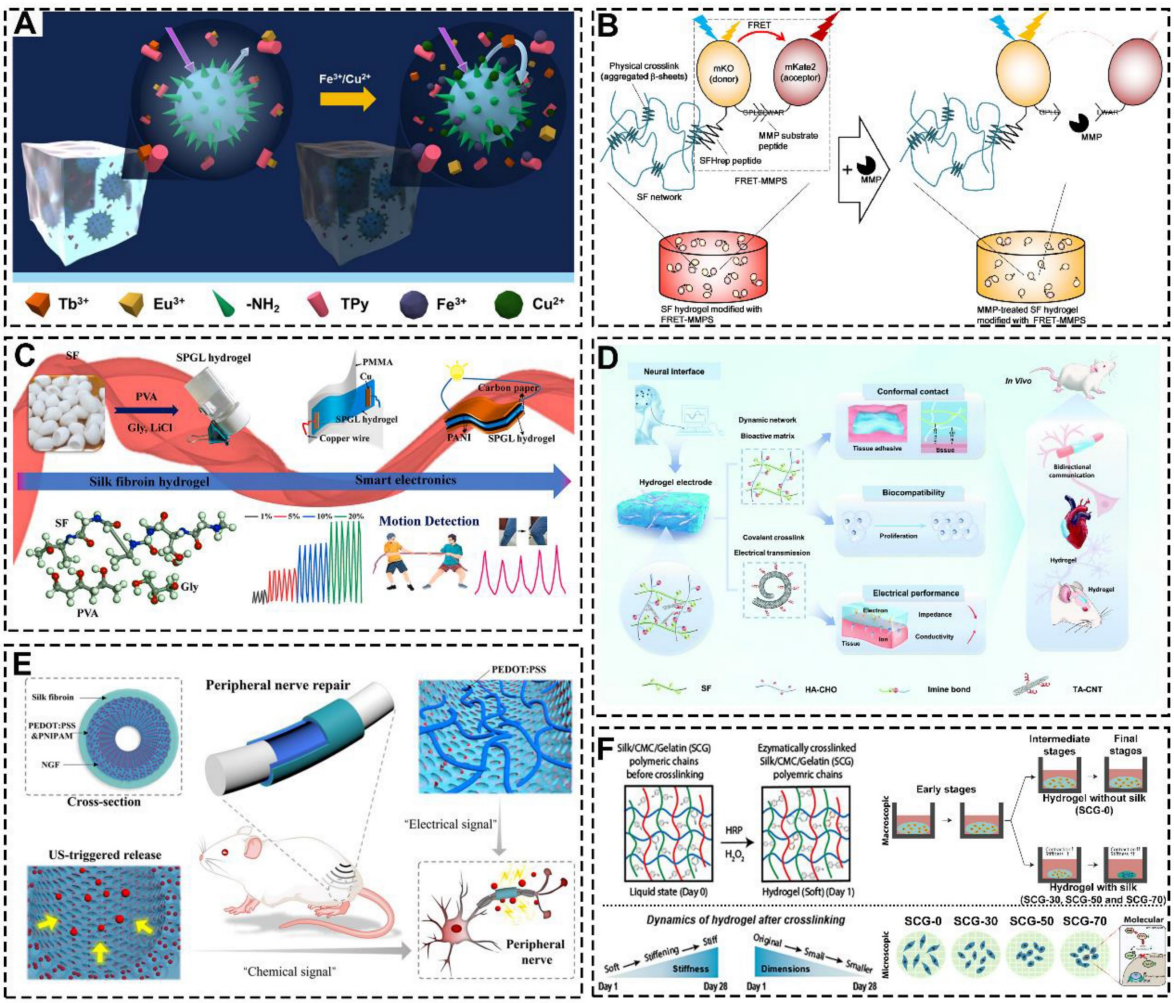
**SF-based hydrogels detect raw biological signal data and provide precision regulation: (A)** The SF-based hydrogel detects metal ions. Reproduced with permission from ref 186; Copyright 2020, Elsevier. **(B)** The SF-based hydrogel detects MMP. Reproduced with permission from ref 187; Copyright 2021, Elsevier. **(C)** The SF-based hydrogel detects stress stimulus. Reproduced with permission from ref 191; Copyright 2023, Elsevier. **(D)** The SF-based hydrogel detects biological electrical signals. Reproduced with permission from ref 188; Copyright 2022, Royal Society of Chemistry. **(E)** The SF-based hydrogel regulated cell behavior through electrical signals. Reproduced with permission from ref 189; Copyright 2023, Wiley-VCH GmbH. **(F)** The SF-based hydrogels regulate chondrogenic differentiation of cells by contracting and stiffening with time contraction Reproduced with permission from ref 190; Copyright 2022, American Chemical Society.

AI for large model computational design requires large amounts of data for model training 185. SF-based hydrogels can be used as biosensors to detect biological signals during the construction of cartilage organoids. For example, Li *et al.* prepared a highly sensitive responsive hydrogel with SF, pectin, polyvinyl alcohol and lanthanide ions (**Figure 12A**) 186. This hydrogel could specifically detect Cu^2+^ and Fe^3+^ ions. Meanwhile, Yamaoka *et al.* prepared a Förster/fluorescence resonance energy transfer (FRET)-based SF hydrogel (**Figure 12B**) 187. This hydrogel could detect the activity of matrix metalloproteinases by FRET signal intensity. Furthermore, SF-based hydrogels can also detect physical signals generated during the construction of cartilage organoids. For example, Feng *et al.* Prepared mechanical detection hydrogels by combining SF with PVA/glycerol/LiCl (**Figure 12C**) 188. In addition, Fan *et al.* constructed SF-HA-CHO hydrogel with dynamic imine crosslinking network (**Figure 12D**) 188. The hydrogels were biocompatible and could detect and record intercellular electrical signals.

AI-driven iterative optimization requires hydrogels with controllable properties, and SF-based hydrogels are well-suited for functional modifications to meet these requirements. For instance, Chai *et al.* prepared SF-based hydrogel conduits by modifying SF with poly(3,4-ethylenedioxythiophene) and poly(4-styrene sulfonate) (**Figure 12E**) 189. The hydrogel conduits achieved controlled drug release by modulating electrical signals. Additionally, Katti *et al.* created ternary network injectable hydrogels using SF, carboxymethyl cellulose, and GT, which showed a time-dependent contraction and stiffening effect that facilitated cartilage regeneration (**Figure 12F**) 190.

## 6. Conclusion and Prospects

As a biomedical material with a long history of applications, SF is considered as an excellent raw material for tissue engineering hydrogels. Here, we reviewed the studies on SF hydrogels on cartilage regeneration in recent years, focusing on the preparation, functionalized modifications and advantages of SF-based hydrogels. Numerous studies demonstrated that SF hydrogels have satisfactory biological properties, suitable internal structure, acceptable mechanical properties, excellent cell-laden ability and remarkable bioactive substance delivery capacity. These features highlight the significant potential of SF hydrogels in cartilage regeneration, repair, and the construction of cartilage organoids.

Despite promising results in preclinical research, SF-based hydrogels remain largely confined to animal experimentation. Challenges persist in translating these hydrogels into clinical applications and constructing cartilage organoids due to their limited adaptability. One major obstacle lies in the inability of SF hydrogels to respond dynamically to cellular processes post-implantation, as they lack intrinsic "intelligence" or the capacity for real-time adjustment. To address these challenges, recent efforts have focused on creating intelligent SF hydrogels. For instance, Baniassadi *et al.* designed magnetic field responsive SF hydrogel with excellent mechanical strength and drugs delivery capability 192. The hydrogel achieved controllable drug release by adjusting the magnetic field. In addition, researchers can attempt to combine SF-based hydrogels with programmable DNA to endow SF-based hydrogels with spatio-temporal regulatory capabilities for fine-tuning the construction of cartilage organoids 137, 193.

**Figure 13:**
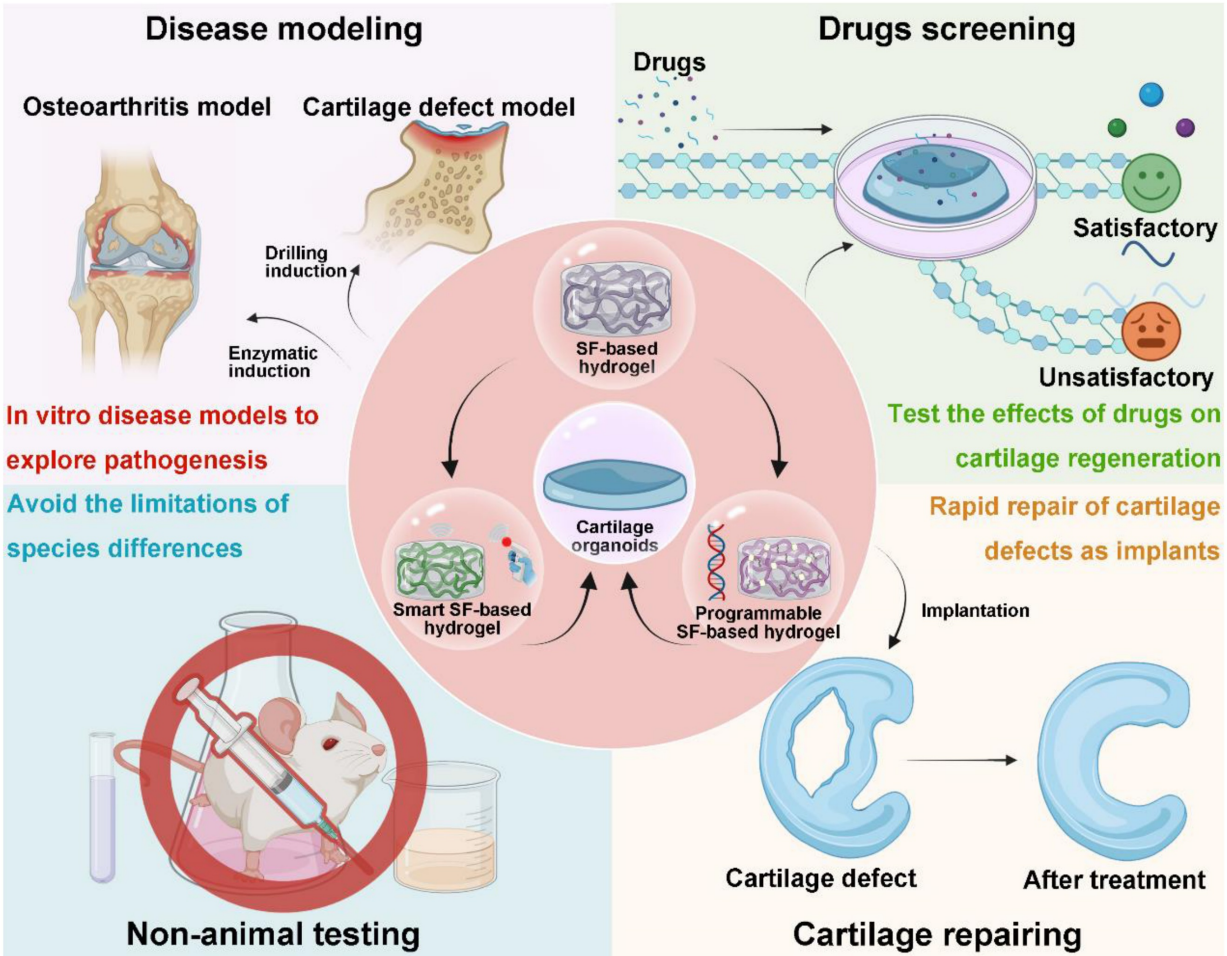
** Development directions of SF-based hydrogels for constructing cartilage organoids: smart SF-based hydrogel and programmable SF-based hydrogel.** Application prospects of cartilage organoids: disease modeling, drugs screening, non-animal testing and cartilage defects repairing. Created with BioRender.com.

Looking forward, SF-based hydrogels offer promising avenues for advancing cartilage research and repair. Key future directions include: (1) Developing Disease Models: Cartilage organoids could be utilized to construct disease models, such as OA)or cartilage defect models, to investigate the underlying mechanisms of disease progression and tissue repair more effectively. (2) Drug Screening and Mechanistic Studies: Organoids offer a valuable platform for drug discovery, enabling researchers to screen compounds (loxoprofen sodium, KGN, D-Glucosamine sulfate, etc.) and explore their effects on cartilage regeneration. (3) Overcoming Clinical Translation Barriers: The species gap between animal models and humans remains a significant hurdle in clinical translation. Using cartilage organoids to evaluate biomaterials for regenerative functions and mechanisms can reduce reliance on animal testing and expedite clinical applications. (4) Cartilage Organoids as Implants: Due to their structural and functional similarity to native cartilage, organoids constructed from SF hydrogels could serve as implantable grafts for cartilage repair, potentially bridging the gap between tissue engineering and clinical practice (**Figure 13**). In conclusion, future efforts to develop smart and programmable SF hydrogels could break through the current bottlenecks in cartilage tissue engineering, offering new tools for modeling disease, discovering therapies, and advancing clinical applications. We believe that SF-based hydrogels are ideal materials for constructing cartilage organoids and can provide new approaches to cartilage regeneration research.

## Figures and Tables

**Table 1:** Comparison of the mechanical properties, degradation, cell viability, and chondrocyte differentiation of SF and other biomaterials used in cartilage tissue engineering.

Property	SF	Col	Alginate	HA	Matrigel
Young's Modulus (MPa)	300-700 MPa	0.1-10 MPa	0.01-1.5 MPa	0.01-0.1 MPa	0.00004-0.00045 MPa
Breaking Elongation (%)	4%-26%	10-30%	10-20%	5-20%	Not reported
Toughness (MJ/m^3^)	70-78 MJ/m3	1-5 MJ/m³	1-5 MJ/m³	1-2 MJ/m³	Not reported
Degradation Time	Tunable (weeks to months)	Days to weeks	Days to weeks	Days to weeks	Within a few days
Cell Viability (%)	>90%	>80%	>80%	>85%	>90%
Chondrocyte Differentiation	Supports viability and promotes collagen type II synthesis	Promotes moderate chondrocyte differentiation but limited stable phenotype	Supports chondrocyte viability with moderate differentiation capacity	Supports viability, requires additional cues for stable phenotype	Supports growth but does not inherently promote chondrocyte differentiation
References	64, 65	66, 67	68-70	71, 72	73-75

**Table 2:** Cross-linking methods for SF-based hydrogels: advantages and limitations.

Type	Cross-linking Method	Advantages	Limitations
Chemical cross-linking	Enzymatic cross-linking	High selectivity; biocompatibility; tunable properties	High cost; low reaction velocity; limited scalability
	Photo-polymerization	Rapid cross-linking; precise control	Potential cytotoxicity; limited tissue penetration
	Cross-linking agents	Cost-effective; enhances mechanical properties	Non-specific reactions; cytotoxicity (for some agents)
Physical cross-linking	Temperature	Non-toxic; simple process	Lacks precision; risk of denaturation at high temperatures
	pH	Effective control over gelation	Requires careful pH control; potential impact on cell viability
	Shear force	Creates directional structures; anisotropic properties	Requires specialized equipment; limited scalability
	Ultrasonication	Non-toxic; controllable process; tailored porosity	Weaker mechanical properties; limited load-bearing capacity
	Electric field	Enables gradient structures; useful for tissue engineering	Requires specialized equipment; potential for uneven cross-linking

**Table 3:** The functional materials that have been used in combination with SF to prepare composite hydrogels for cartilage repair.

Type	Functional materials	Preparation method	Advantages of composite hydrogels in cartilage repair	References
Synthetic	GO	Photo-polymerization with RuBPY as photo-initiators	Excellent mechanical properties	112, 113
	PEG	Ultrasonication	Injectable; rapid gelation; suitable microenvironment	114
	PEGDMA	UV photo-polymerization	Adjustable mechanical properties	115
	PEGDA	Photo-polymerization with LAP as photo-initiators	Excellent mechanical properties; bioprinting-compatible	116
	poly(N-vinylcaprolactam)	Photo-polymerization with tris(2,2-bipyridyl)dichlororuthenium(II) and ammonium peroxodisulfate as photo-initiators	Enhanced water uptake capacity, elasticity and toughness	117
	PVA	pH adjustment	Excellent mechanical properties; high porosity; adjustable swelling ratio	118
	POL	Enzyme cross-linking catalyzed by HRP and H_2_O_2_	Thermosensitive; injectable	119, 120
	MXene	Enzyme cross-linking catalyzed by HRP and H_2_O_2;_ Ultrasound technique	Metallic conductivity; piezoelectricity; excellent hydrophilicity; diverse surface chemical properties; injectable	121, 122
Natural	Collagen	Ultrasonication	Promotes MSC proliferation; chondrogenic differentiation	123, 124
	Gelatin	Enzyme cross-linking catalyzed by transglutaminase	Cell attachment, proliferation; excellent mechanical integrity	125, 126
	HPMC	Heating	Excellent mechanical properties	127
	CS	Enzyme cross-linking catalyzed by HRP and H_2_O_2_	Environmentally sensitive; controlled release of drugs and growth factors	128-131
	HA	Enzyme cross-linking catalyzed by HRP and H_2_O_2_	MSC recruitment; cell adhesion; cartilage-like mechanical properties	132-135
	GA	Photo-polymerization	Reduced mechanical stress; lower *in vivo* friction coefficients	136
	DNA	Enzyme cross-linking catalyzed by HRP and H_2_O_2_ and complementary base pairing	Regulate chondrogenic differentiation of BMSCs	137

## Abbreviations

OA: osteoarthritis
ECM: extracellular matrix
SF: silk fibroin
AI: artificial intelligence
ACI: autologous chondrocyte implantation
MACI: mx-induced autologous chondrocyte implantation
MSCs: msenchymal stem cells
COs: callus organoids
HRP: horseradish peroxidase
TA: tyramine
GT: gelatin
SF C-gel: SF hydrogels by using Co-60 derived gamma-ray
GTA: glutaraldehyde
SF/PCZ: polycarbazole/silk fibroin hydrogels
KGN: kartogenin
PDGF-BB: platelet-derived growth factor BB
BSNF: beta-sheet rich silk nanofibers
BMSCs: bone marrow mesenchymal stem cells
RSF: regenerated silk fibroin
PEG: polyethylene glycol
PEGDMA: poly(ethylene glycol) dimethacrylate
SilMA: silk methacrylate
DN: double network
ALG-POL: alginate-poloxamer
Col: collagen
HA: hyaluronic acid
GA: glycyrrhizic acid
IPN: interpenetrating network
HPMC: hydroxypropyl methyl cellulose
CS: chitosan
PC: propanoic acid-modified chitosan
DNA: deoxyribonucleic acid
HG-SF: host-guest interactions silk fibroin
GelMA: gelatin methacrylate
Silk-GMA: SF-glycidyl-methacrylate
AD/CS/RSF/EXO: alginate-dopamine/chondroitin sulfate/regenerated silk fibroin/exosome hydrogel
BBR: berberine
FRET: förster/fluorescence resonance energy transfer
